# Integration of the Microbiome, Metabolome and Transcriptomics Data Identified Novel Metabolic Pathway Regulation in Colorectal Cancer

**DOI:** 10.3390/ijms22115763

**Published:** 2021-05-28

**Authors:** Vartika Bisht, Katrina Nash, Yuanwei Xu, Prasoon Agarwal, Sofie Bosch, Georgios V. Gkoutos, Animesh Acharjee

**Affiliations:** 1 Institute of Cancer and Genomic Sciences, College of Medical and Dental Sciences, University of Birmingham, Birmingham B15 2TH, UK; vartikabisht6197@gmail.com (V.B.); y.xu@bham.ac.uk (Y.X.); g.gkoutos@bham.ac.uk (G.V.G.); 2 MRC Health Data Research UK (HDR UK), Midlands B15 2TT, UK; 3 College of Medical and Dental Sciences, University of Birmingham, Birmingham B15 2TT, UK; katrinanash649@outlook.com; 4 Institute of Translational Medicine, University Hospitals Birmingham NHS, Foundation Trust, Birmingham B15 2TT, UK; 5 KTH Royal Institute of Technology, School of Electrical Engineering and Computer Science, 100 44 Stockholm, Sweden; prasoonagar@gmail.com; 6 Science for Life Laboratory, 171 65 Solna, Sweden; 7 Department of Gastroenterology and Hepatology, AG&M research institute, Amsterdam UMC, 1105 AZ Amsterdam, The Netherlands; bosch.sofie@gmail.com; 8 NIHR Surgical Reconstruction and Microbiology Research Centre, University Hospital Birmingham, Birmingham B15 2WB, UK; 9 NIHR Experimental Cancer Medicine Centre, Birmingham B15 2TT, UK; 10 NIHR Biomedical Research Centre, University Hospital Birmingham, Birmingham B15 2TT, UK

**Keywords:** microbiota, colorectal neoplasms, biomarkers, metabolomics, transcriptome, omics integration

## Abstract

Integrative multiomics data analysis provides a unique opportunity for the mechanistic understanding of colorectal cancer (CRC) in addition to the identification of potential novel therapeutic targets. In this study, we used public omics data sets to investigate potential associations between microbiome, metabolome, bulk transcriptomics and single cell RNA sequencing datasets. We identified multiple potential interactions, for example 5-aminovalerate interacting with *Adlercreutzia*; cholesteryl ester interacting with bacterial genera *Staphylococcus*, *Blautia* and *Roseburia*. Using public single cell and bulk RNA sequencing, we identified 17 overlapping genes involved in epithelial cell pathways, with particular significance of the oxidative phosphorylation pathway and the *ACAT1* gene that indirectly regulates the esterification of cholesterol. These findings demonstrate that the integration of multiomics data sets from diverse populations can help us in untangling the colorectal cancer pathogenesis as well as postulate the disease pathology mechanisms and therapeutic targets.

## 1. Introduction

Colorectal cancer (CRC) is the second most common cause of death due to cancer worldwide, with an incidence of almost two million cases in 2018 [1]. Early detection and treatment are critical factors in the course and prognosis of CRC, as the survival rate decreases with disease progression [2]. It is thought that CRC arises due to complex interactions of the transcriptome, metabolome, microbiome and immune system [3]. Assessment of these omics platforms and their associations may reveal important pathways that can be used for early cancer detection as well as therapeutic targets. Recent development of high throughput sequencing technology has enabled the quantification of the expression labels of multiple omics data including metabolomics, transcriptomics microbiome and inflammatory markers.

Growing evidence indicates that the gastrointestinal microbiome is strongly associated with the development of colorectal cancer, as abundance of specific microbiota have been found to be increased or decreased in colorectal cancer patients in comparison to healthy controls [4]. A recent study, by Ternes et al. (2020), collated all current knowledge on the gut microbiome in colorectal cancer; *Fusobacterium*, *Peptostreptococcus*, *Porphromonas*, *Prevotella*, *Parvimonas*, *Bacteroides* and *Gemella* were identified to be the most prominent CRC-associated bacteria [4].

The human and microbial metabolome may also play a vital role in colorectal carcinogenesis [5]. For example, polyamines, which are thought to contribute to carcinogenesis, show a higher abundance in the CRC patients in comparison to healthy individuals [5]. Conversely, poly- and monounsaturated fatty acids, short chain fatty acids and hydrocinnamic acid have been found to be decreased in multiple CRC cohorts [5].

Within the transcriptomics realm, it has been estimated that 10% of the human epithelium transcriptome is regulated by the gut microbiome via the metabolome [6]. The biological mechanisms for this relationship are not fully understood, though there are multiple ideas theorising this relationship, as the majority of these genes are involved in immunity, cell proliferation and metabolic pathways [6].

There are very few studies that explored the interplay between different sets of microbiome, metabolome and transcriptomics datasets. Wang et al. recently identified significant associations between the microbiome, butyrate-related metabolites and gene expression in patients with CRC [7]. However, this was a pilot study which used only four samples to identify relationships between metabolites, microbial taxa and DNA methylation data sets. Other studies solely focused on single or two omics data sets. For example, Clos Garcia performed an integrative analysis of faecal UHPLC-MS metagenomics and 16S metagenomics in CRC in 224 faeces samples [7]. Differences in faecal levels of cholesteryl esters and sphingolipids were identified. Additionally, *Fusobacterium*, *Parvimonas* and *Staphylococcus* were increased in CRC whilst the *Lachnospiraceae* family were reduced. Integration of this data identified tight interactions which were superior to the conventional faecal occult blood test for CRC diagnostics. Similarly, Kim et al. identified strong gut microbiome–metabolome associations in CRC and colorectal adenoma patients, supporting the importance of metabolites and their interplay in the development of CRC [8]. Data on clinical measurements, gene expression, DNA methylation and miRNA expression have also been integrated to identify a prognostic model, which was shown to improve prediction of prognosis in CRC patients [9].

Exploration of the relationship between the microbiome, metabolome and transcriptomics is vital to improve CRC prevention, diagnosis and treatment. Integration of multiomics data sets may provide knowledge on CRC pathogenesis, in addition to which bacteria may drive or protect against carcinogenesis. This could enable targeted probiotic administration, antibiotic treatment, or nutritional interventions to alter the gastrointestinal microbiome to prevent or treat CRC [10]. Metabolites could also be administered if they are known to protect against, or halt progression, of CRC.

In this study we used public omics data sets to investigate the association between the microbiome, metabolome, bulk transcriptomics and single cell RNA sequencing datasets, aiming to explore their potential role in colorectal cancer pathogenesis and to postulate causative mechanisms for associations identified.

## 2. Results

### 2.1. Microbiome and Metabolome Analysis Identified Novel Interactions

In order to identify potential microbiome–metabolome interactions, we applied a Bayesian additive regression trees classification method (BART) to discriminate CRC cases from healthy controls in two published datasets; Kim et al. [8] and Clos-Garcia et al. [11]. The BART method was chosen because it is capable of both capturing nonlinear effects and estimating uncertainties associated with the estimates. Models were built with (1) microbiome only and (2) combined microbiome and metabolome data. In each case, two steps were followed. First, BART was used to select important features by local thresholding (please see Section 4 for more details). Second, having obtained the set of important features, a cross-validated BART model was built to optimise the classification accuracy. Since, within Kim et al. [8] dataset, controls (*n* = 102) were over-represented in relation to cases (*n* = 36), step 1 was repeated 10 times, each time using a different, but balanced, data resampling, in order to reduce the bias introduced by subsampling. Hypothesising that interactions that appear more frequently are potentially interacting ones, potential interaction effects were examined by counting the number of times each pair of features co-occurred in any downward path of the fitted trees. Partial dependence plots were constructed for both individual features and high frequency interacting pairs.

**Figure 1:**
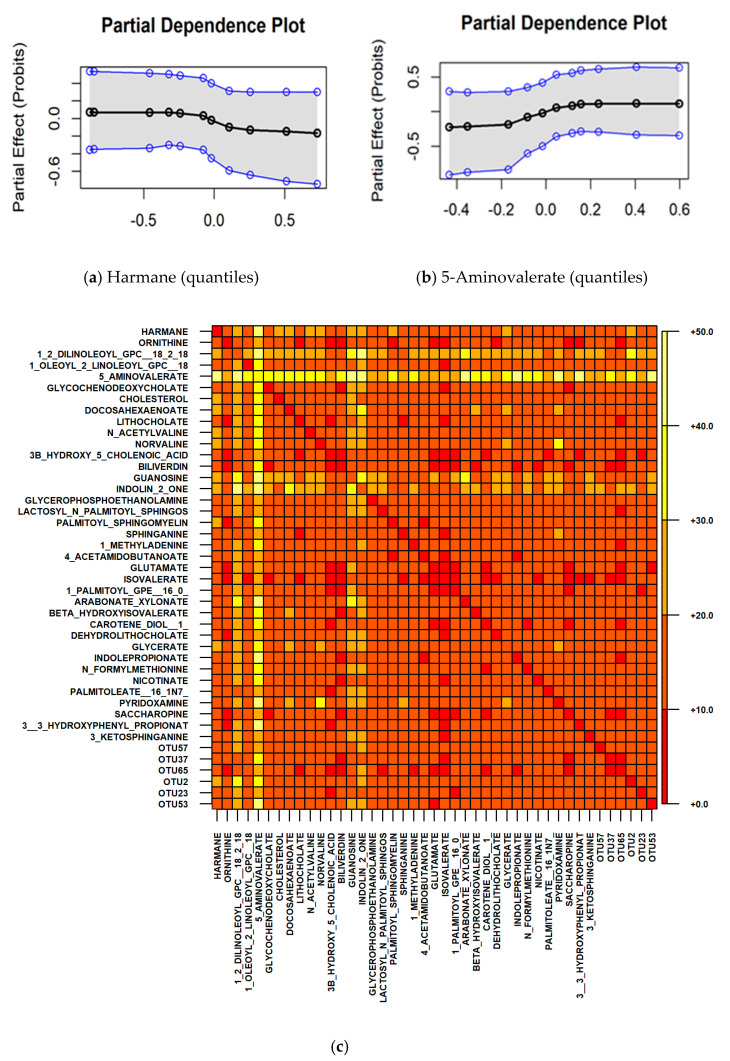
(**a**) Results from Kim et al. microbiome and metabolome analysis using BART methods; (**a**) Bayesian Additive Regression Trees (BART) model partial dependence plots for harmane, plotted at various quantiles (*x*-axis). *y*-axis shows the probits, a value of 0 indicates that CRC and normal are equally likely; values above 0 indicate that CRC is more likely and values below 0 indicate CRC is less likely. The shaded area shows the 0.95 Bayesian credible intervals of the probits; (**b**) BART model partial dependence plots for 5-aminovalerate, plotted at various quantiles (*x*-axis). *y*-axis shows the probits, a value of 0 indicates that CRC and normal are equally likely; values above 0 indicate that CRC is more likely and values below 0 indicate CRC is less likely. The shaded area shows the 0.95 Bayesian credible intervals of the probits; (**c**) matrix of counts of pairwise interactions from the BART mode, shown in the heatmap. The genera are *Veillonella* (OTU57), *Eubacterium* (OTU37), *Haemophilus* (OTU65), *Adlercreutzia* (OTU2), *Anaerotruncus* (OTU23), and *SMB53* (OTU53). The metabolic and microbiome features are separated.

Both microbiome and metabolome features, within the Kim et al. [8] dataset, were selected (Supplementary Table S2). The out-of-sample (oos) misclassification error of the cross-validated BART model was 0.306. When only microbiome features were used, however, the oos error increased to 0.528, implying that the model, built on microbiome features alone, does not convey predictive power in differentiating carcinoma from control samples. Figure 1a,b shows the partial dependence of harmane and 5-aminovalerate, two of the metabolic features that were selected in all 10 repeats. We observe a decrease in the risk of cancer in relation to an increase of harmane levels, in addition, the risk is elevated as 5-aminovalerate increases. Partial dependence plots for the rest of the features, which were selected in at least half of the runs, are presented in Supplementary Figure S1. Although these features are selected by the model as significantly impacting the prediction of the outcome, no strong relationship with CRC risk was observed when considered individually, as illustrated, for example, by the partial dependence plots of ornithine, 1,2-dilinoleoyl-GPC (18:2/18:2), glycochenodeoxycholate, cholesterol and bacterium genus *Eubacterium* (OTU37). However, this inconsistency may be explained by interaction effects, which are not captured by partial dependence, since partial dependence, by definition, averages out the effect of all other variables.

We therefore investigated pairwise interaction effects for all selected microbiome and metabolomic features. Figure 1c displays the interaction count matrix resulting from the BART model. The most noticeable metabolite is 5-aminovalerate, which is shown to interact with many other metabolites and some bacteria, in particular, the genus *Eubacterium*, *Adlercreutzia* and *SMB53*. Although less prominent, 1,2-Dilinoleoyl-GPC (18:2/18:2), guanosine and indolin-2-one also were shown to be involved in multiple interactions.

These metabolites were shown to interact with 5-aminovalerate as well amongst themselves, as indicated by the interactions among all such pairs highlighted in Figure 1c. This suggests that high-order interactions may be present.

**Figure 2:**
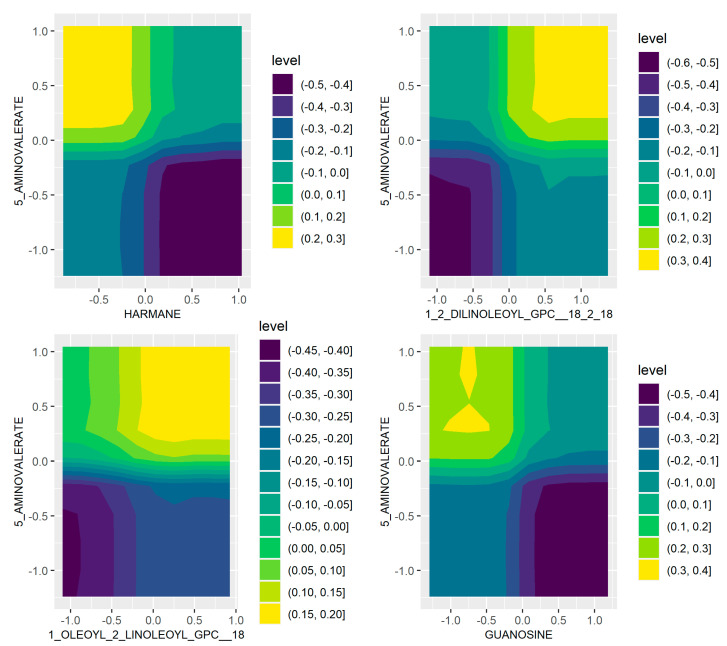
Partial dependence on CRC for representative pairs of microbial/metabolomic features, shown as contour plots, coloured by log-odds of CRC. Higher values (yellow) indicate increased risk of CRC; lower values (blue) indicate decreased risk of CRC; a value of 0 indicates the risks are equal. Plots for more feature pairs can be found in Supplementary Figure S2. Top to bottom: (5-aminovalerate, harmane), (5-aminovalerate, 1,2-Dilinoleoyl-GPC), (5-aminovalerate, 1-oleoyl-2-linoleoyl-GPC), (guanosine, 5-aminovalerate).

The top 2% feature pairs with the highest counts in Figure 1c were selected for partial dependence investigation. For each such pair, we estimate log-odds of cancer on a regular 2D grid of feature values of this pair, by fixing the feature values to be on the grid and averaging over all predictions from the training instances. Among all feature pairs, higher risk of CRC implies elevated level of 5-aminovalerate, however, increased 5-aminovalerate does not necessarily imply higher risk of CRC, when considering the effect of other metabolites (Figure 2 and Supplementary Figure S2). In particular, increased harmane, guanosine, arabonate_xylonate, glycerate, N_Formylmethionine and Pyridoxamine levels or decreased 1,2-Dilinoleoyl-GPC (18:2/18:2), N_Acetylvaline and indolin-2-one levels, are shown to lower the risk of CRC, even though 5-aminovalerate is abundant. When 5-aminovalerate is not abundant, CRC risk is minimised, for example, by increasing harmane, guanosine, or by decreasing 1,2-Dilinoleoyl-GPC (18:2/18:2) levels. In addition, the absence of the genus *Adlercreutzia* is shown to lower the risk of CRC when 5-aminovalerate is elevated. The complete results are represented within Supplementary Table S3.

Although when not considering interaction effects, CRC risk increases as 5-aminovalerate levels increase (Figure 1b), the result of BART interaction analysis suggests that the risk is attenuated when controlling for other metabolites. This observation offers additional insights to the regulation of metabolomic and microbiome markers in CRC tumorigenesis, and could serve as a basis for future experimental and validation studies in the search for potential targets for therapeutic intervention.

**Figure 3:**
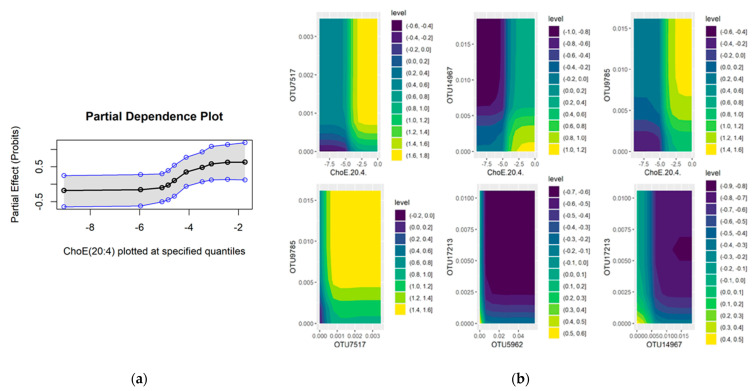
Partial results for Clos-Garcia microbiome and metabolome analysis using BART methods; (**a**) partial dependence plot of ChoE(20:4); (**b**) contour plots for microbiome–metabolome interactions between (1) *Staphylococcus* (OTU7517) and ChoE(20:4), (2) *Blautia* (OTU14967) and ChoE(20:4), (3) *Roseburia* (OTU9785) and ChoE(20:4); microbial interactions between (1) *Roseburia* (OTU9785) and *Staphylococcus* (OTU7517), (2) *Blautia* (OTU5962) and an unknown genus from family *Lachnospiraceae* (OTU17213), (3) OTU17213 and *Blautia* (OTU14967). The *z*-value (level) is the log-odds of CRC, interpreted in the same way as Figure 2.

The Clos-Garcia [11] dataset analysis revealed a better performance in discriminating CRC from healthy controls, in both combined microbiome metabolome dataset (oos: 0.094) and microbiome dataset alone (oos: 0.113). Compared with Kim et al. [8], the Clos-Garcia [11] microbiome samples confer considerably higher predictive power. This noticeable difference in the classification error between the two published datasets may be partly explained by the observation that no common microbes, at the genus level, were selected by BART. The BART interaction analysis identified three microbiome–metabolome pairs and three microbial pairs that exhibit strong interactions compared to other feature pairs. All microbiome–metabolome pairs involve ChoE(20:4), which is shown to interact with the bacteria genera *Staphylococcus*, *Blautia* and *Roseburia*. Partial dependence of ChoE(20:4) showed that the risk of CRC increases when ChoE(20:4) is high, although the relation is nonlinear as the risk remains low as long as ChoE(20:4) level is below some threshold (Figure 3a). Partial dependence of these feature pairs (Figure 3b), however, provides us with valuable insights into how the risk may be mitigated by controlling for specific microbes. As can be seen from the contour plots, under high values (log-normalised) of ChoE(20:4), decreasing *Staphylococcus* and *Roseburia*, or increasing *Blautia*, reduces the risk of CRC. Further inspection of the contour plots for microbial interactions reveals that the risk is minimised when *Staphylococcus* and *Roseburia* decrease simultaneously; and when *Blautia* and an unknown genus (OTU17213), from the same family as *Blautia*, *Lachnospiraceae*, increase simultaneously. Supplementary Table S4 provides a summary of these results.

### 2.2. Metabolite Enrichment Analysis

The Kim et al. [8] metabolites dataset was also analysed using the IMPaLA (Integrated Molecular Pathway Level Analysis) tool. IMPaLA is a web tool, developed for integrated pathway analysis of metabolomics data alongside gene expression or protein abundance data [12,13]. It works through extending over-representation and enrichment analyses to multiple data types. Supplementary Table S5 depicts the pathways enriched with the Kim et al. [8] metabolites. The ABC transporters and Purine Nucleoside Phosphorylase pathways are the most significant pathways associated with the Kim et al. [8] metabolites, KEGG (*p* value: 6.9 × 10^−10^) and SMPDB (*p* value: 7.65 × 10^−8^), respectively.

Supplementary Table S6 shows the list of IMPaLA identified enriched pathways using the Clos-Garcia metabolite dataset. Amongst the most significantly enriched pathways were the ABC transporter (*p* value: 6.09 × 10^−9^) and the Purine Nucleoside Phosphorylase Deficiency (*p* value: 7.65 × 10^−8^) pathways. Cholesterol was found to be significantly enriched among several biochemical and metabolic pathways, primarily featuring pathways involving vitamin digestion and absorption (*p* value: 0.0003), and digestion of dietary lipids (*p* value: 0.0006).

### 2.3. Bulk RNA Seq Analysis

**Figure 4:**
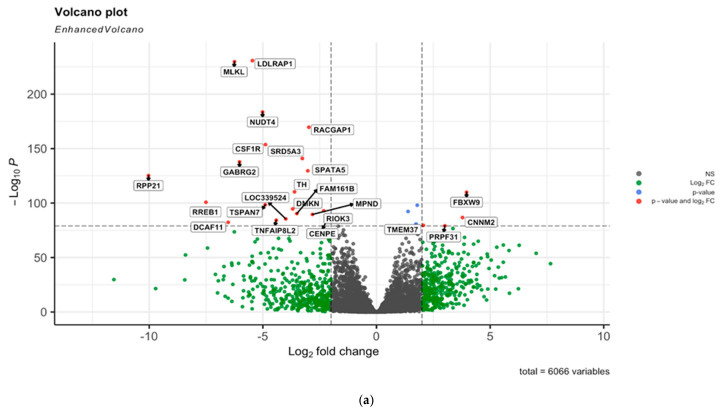

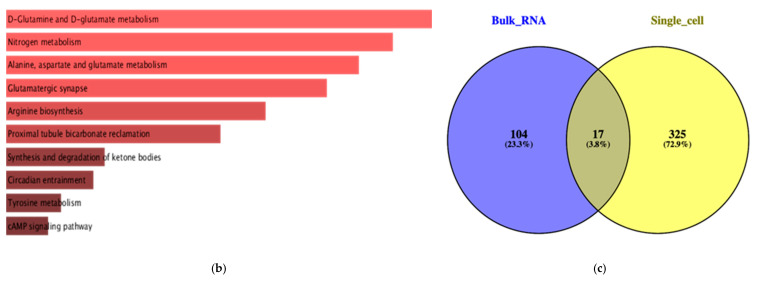
(**a**) Volcano plot for 6066 genes selected after preprocessing with *p* adjusted cut-off 10e-100 shown. Significantly up- or downregulated genes are shown in red; (**b**) enrichment analysis of the 76 genes participating in pathways that are presented in barplot. (**c**) Venn diagram representation of the common genes between bulk RNAseq analysis and single-cell analysis. In total 17 genes were found common between the RNA and single cell datasets.

A list of 6066 differential genes were obtained from COAD—TCGA (The Cancer Genome Atlas—Colon Adenocarcinoma) [14] study after iterative undersampling, detailed in Section 2, and the resulting *p* value significance of the fold change was 0.0001. Out of the 6066 genes, 2653 (43.74%) were upregulated and 3413 (56.26%) were downregulated. Figure 4a shows a volcano plot for the selected 6066 genes. The application of a Recursive Feature Elimination [15] over the 6066 differential gene expression dataset resulted in the identification of 121 unique genes. Out of these 121 genes, 76 appear more than once among the iterations. We used Enrichr [16] for the enrichment analysis for the selected 76 genes which revealed that the Glutamatergic synapse pathway was significantly associated (*p* value: 0.001) with the *GNG3*, *GLS2*, *GNG7*, *GRIK1*, *GRIA3* and *GRIN2D* genes (Figure 4b). Furthermore, the alanine, aspartate and glutamate metabolism pathways were significantly associated (*p* value: 0.02) with *GLS2*, *GPT* and *ASPA*. A list of KEGG pathways can be found in Supplementary Table S1.

### 2.4. Single Cell RNA Seq Analysis

We obtained four lists of differential genes in four different cell types described by Li H et al. (2017) [17] listed in Supplementary Table S7. DAVID enrichment analysis was performed to obtain the significant pathways for these genes [18]. For the case of fibroblast cells associated dataset, the most significant pathway obtained was Proteoglycans in cancer (*p* value 1.2 × 10^−3^) (*CD63*, *DDX5*, *DCN*, *LUM*, *ITGB1*). In epithelial cells, oxidative phosphorylation was identified as the most significant pathway (*p* value 2.0 × 10^−6^) (*ATP5A1*, *ATP5G1*, *ATP5I*, *NDUFB1*, *COX6C*, *COX7C*, *UQCR10*). In case of myeloid cells, the key pathways identified were the oxidative phosphorylation (*p* value 2.2 × 10^−3^) (*ATP6*, *ATP8*, *ND6*, *ND8*) as well as metabolic pathways (*p* value 8.6 × 10^−2^) (*ATP6*, *ATP8*, *ND6*, *ND8*, *CECR1*, *SAT1*). For the B-cell related dataset, the most significant pathway identified was the protein processing in the endoplasmic reticulum (*p* value 4.9 × 10^−2^) (*HSPA8*, *HSPH1*) pathway. The 342 genes and pathways identified within the epithelial cells related dataset (obtained from Zhang GL et al. (2019) [19]) are provided within the Supplementary Table S8.

**Figure 5:**
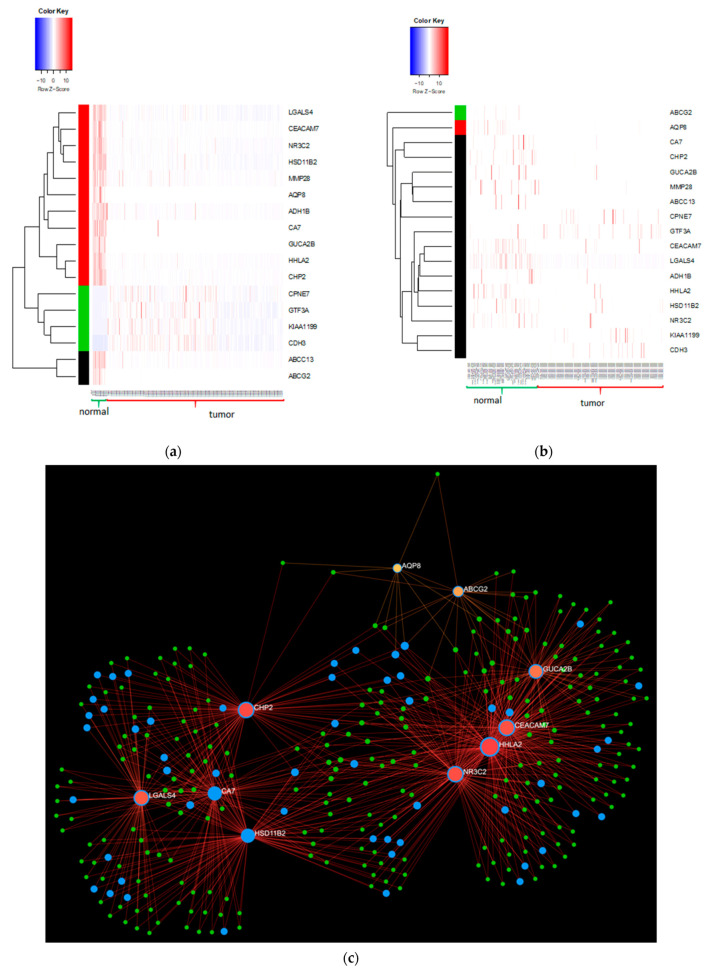
(**a**) Shows the heatmaps for the 17 genes in the bulk RNA sequencing dataset; (**b**) heatmap for the 17 genes (single cell RNA sequencing). Red denotes upregulation, white indicates no expression or zero expression and blue depicts low expression; both heatmaps were performed using hierarchical clustering on the rows. The three clusters are coloured as red, black and green; (**c**) the network was generated using the 17 genes that were found to be differentially expressed in the network. The seed genes are labelled and shown in red, while the proteins connected to these genes are shown in green. The interactions between the genes and the proteins are shown in red. The blue coloured proteins or seed genes are involved in the metabolic pathways from KEGG.

Significant downregulated pathways identified were the oxidative phosphorylation (*p* value 0.000347) (*NDUFB1*, *COX6B1*, *COX7A2*, *COX7C*, *ATP5C1*, *ATP5G1*, *ATP5H*) and the nitrogen metabolism (*p* value 0.000595) (*CA1*, *CA7*, *CA2*) pathways. The p53 signalling pathway (*p* value 0.00244) (*ATR*, *MDM2*, *PERP*, *SESN3*) was depicted as significantly upregulated. We further compared the 121 significant genes obtained from the bulk RNA-seq analysis and compared them to the 342 genes obtained from single-cell epithelial cells. A total of 17 genes were identified to be present in both analyses (Figure 4c). All 17 genes show a similar regulatory pattern in both the bulk and single-cell RNA-seq and are represented in Table 1. A heatmap for the 17 genes reveals a differential gene expression pattern across both the bulk (Figure 5a) and single cell RNA seq datasets (Figure 5b). We then employed the Network Analyst platform [20] and constructed a tissue specific interaction network using these 17 genes (Figure 5c). In the resulting network the seed nodes are shown in red while the genes that are involved in metabolism are shown in blue.

### 2.5. Qualitative Integration

**Figure 6:**
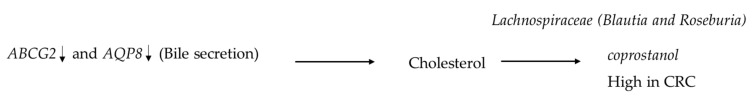
*ABCG2* and *AQP8* genes are responsible for bile secretion and bile is converted to cholesterol by the gut enzymes [63]. Cholesterol is further converted to *coprostanol* by members of the *Lachnospiraceae* family [64].

We attempt to integrate multiple features from diverse data sets based on the enrichment analysis and literature review. *ABCG2* and *AQP8* genes are responsible for bile secretion (AQP8 is downregulated), which is converted to cholesterol by the gut enzymes[63]. Cholesterol is further converted to coprostanol by members of the *Lachnospiraceae* family, including *Blautia* and *Roseburia* [64]. In Figure 6, we provide an example of the qualitative integration.

## 3. Discussion

In this study, we used multiomics data sets from different populations to understand pathophysiological pathways and identify potential therapeutic CRC targets. In an effort to understand the complex interactions between microbiome and metabolome we employed two datasets (Kim et al. [8] and Clos-Garcia [11]) and quantified the top ranked interactions using the BART model. We observed a decrease in the predictive risk of CRC as the level of harmane increased with estimated credible intervals from BART. In our BART model, sex and age were used as a covariate. Kim et al. also considered age, sex, and smoking status (smoker/non-smoker) as covariates. Moreover, we were able to show that this predictive risk is elevated as serum levels of faecal 5-aminovalerate increases. In this case, 5-aminovalerate are considered as interactions as they appear in a contiguous downward path of the tree from the root node to a terminal node. Moreover, a partial dependence of ChoE(20:4) showed that the risk of CRC increases when cholesteryl ester, ChoE(20:4), is high. Importantly, a pairwise interaction analysis suggests that the risk may be mitigated by controlling for other microbial and/or metabolomic features, as shown in Supplementary Tables S3 and S4. This offers great potential for targeted experimental pathophysiology studies which could provide further evidence for our findings. To make our analysis consistent across the two datasets of Kim et al. and Clos-Garcia et al., we removed the adenoma samples and considered only the carcinoma and healthy controls, instead of pooling adenomas and carcinomas together. Clos-Garcia et al. identified increased levels of *Fusobacterium*, *Parvimonas* and *Staphylococcus*, with decreased levels of *Lachnospiracaea* family members in CRC [11]. We also note that in Clos-Garcia et al., a logistic regression model was only fitted to 16 genera and 6 metabolites, which were identified by comparison with their previously published results. Conversely, in our case all available microbiome and metabolome features were included in BART modelling. Kim et al. identified multiple associations between bacteria and metabolites [8].

To add biological pathway information on the metabolomics and microbiome data sets, we used enrichment analysis. Interestingly, based on an analysis of the Kim et al. metabolomics dataset, we identified many biochemical pathways enriched with 5-aminovalerate. We found 5-aminovalerate to be significantly enriched within the arginine and proline metabolism (*p*-value: 0.00243) and D-arginine and D-ornithine metabolism (*p* value: 0.0541) pathways. Lysine, proline and arginine have previously shown antitumour effects on cancer cell line HCT 116 by inhibiting *MMP* expression and invasion [65]. Although it may be thought that their metabolism would be downregulated in cancer if a tumour has been able to develop, a systematic review evaluating the effects of arginine on colorectal cancer found decreased tumour production and crypt cell hyperproliferation during the initiation stage of carcinogenesis, but a stimulation of tumour growth during the promotion stage [66]. Evidence also indicates that the gut microbiome plays an important role in the production of 5-aminovalerate. Lower levels of 5-aminovalerate are found in germ free mice in comparison to normal mice [67,68], and high levels are thought to indicate bacterial overgrowth [69]. In addition to that, 5-aminovalerate is thought to be a metabolite of dietary proteins, particularly lysine, produced by bacteria in the gastrointestinal tract and has also been found to be increased in a haem-enriched diet, which has also been associated with a higher risk of CRC [70].

A metabolic pathway enrichment analysis also identified the short chain fatty acids (SCFAs) acetate, propionate and butyrate to be significantly associated (*p* < 0.05) with CRC. However, in the data set the expression label was low, so it might be due to the quantification of the metabolomics experimental process. Fibre is digested by anaerobic bacteria in the gut to produce these SCFAs [71]. SCFAs have been detected in higher levels in healthy individuals in comparison to CRC patients, and have been shown to inhibit histone deacetylase activity and interact with cell surface receptors in enterocytes affecting the epigenetic gene expression [72]. Their role is postulated to be protective against CRC, as they have been shown to arrest growth and differentiation in human colon carcinoma cells [73].

In our analysis we performed an integration of metabolomic, microbiome and transcriptome datasets from multiple different CRC cohort studies to identify potential new disease targets. Methodologically it is different from Acharjee et al. [73], and Quraishi et al. [74]. In both of these studies, omics integration was performed from the same cohorts for obesity and inflammatory bowel disease. The data integration performed here for different CRC patient cohorts is being done for the first time. However, in the case of humanised microbiome mouse models [75] the transcriptome, microbiome and metabolites were integrated.

In this analysis, we identified 17 genes common between bulk and single cell RNA sequencing datasets and these genes were used for the pathway analysis. Interestingly several genes like *KIAA1199*, *CDH3*, *GUCA2B*, *LGALS4*, *CA7*, *NR3C2*, *ABCG2*, *AQP8*, etc. were found to be implicated in CRC pathophysiology. Few genes that we have identified are involved in various metabolic processes, for example *HSD11B2* (converts cortisone to cortisol), *NR3C2* and *HSD11B2* (Aldosterone regulated sodium reabsorption), *ABCG2* and *AQP8* (Bile secretion), *CA7* (nitrogen metabolism). In our metabolic analysis we identified choE(20:4), also known as cholesteryl ester, as a significant metabolite. It is synthesised by the esterification of cholesterol; the enzyme responsible for it is *ACAT1* [76]. Our network analysis suggests that *ACAT1* and several other metabolic genes are interacting with the 17 genes we identified either directly or indirectly. Metabolites with high ranking seem to be interacting with multiple microbiome species, for example Indolin-2-one is a tryptophan metabolite, likely of gut microbiome origin [77]. Indolin-2-one compounds have been identified as an effective cancer treatment, with indolin-2-one derivatives showing anticancer effects in vitro against ovarian cancer cells [78,79,80]. Louis et al. [81] have also identified higher log blood harmane concentration in colon cancer and other cancers in comparison to those without cancer.

There are some limitations of this study. Firstly, the different cohorts of populations employed exhibit heterogeneity which limits comparability of results. Secondly, we only considered pairwise interactions. However, it is anticipated that higher order interactions may be present, as indicated by all pairwise interactions between *Staphylococcus*, *Roseburia* and ChoE(20:4) (Cholesterol Ester). Thirdly, nutrition, exercise and other lifestyle factors strongly influence the composition of the gut microbiome and metabolome. Unfortunately, these could not be considered in our analysis. Fourthly, the targets (metabolites, genes and microbiota) generated from this study were not validated in follow-up for the participants as only one stool sample was collected. Potential future studies could include the collection of multiple longitudinal stool samples for causal analysis to improve validity of results.

A small proportion of colorectal cancers, which are not included in our analysis, are directly caused by inherited gene mutations, including mutations of the *MUTYH* gene or mismatch repair genes [82]. Nonetheless, alterations in gene expression are seen across all patients with colorectal cancer [82]. Mutations in the *APC*, *Kirsten-ras* and *p53* genes are thought to be an alternative pathway in tumour development [83]. Additionally, the transcription of other genes may be upregulated or downregulated in CRC [84], as seen in our analysis.

## 4. Materials and Methods

**Figure 7:**
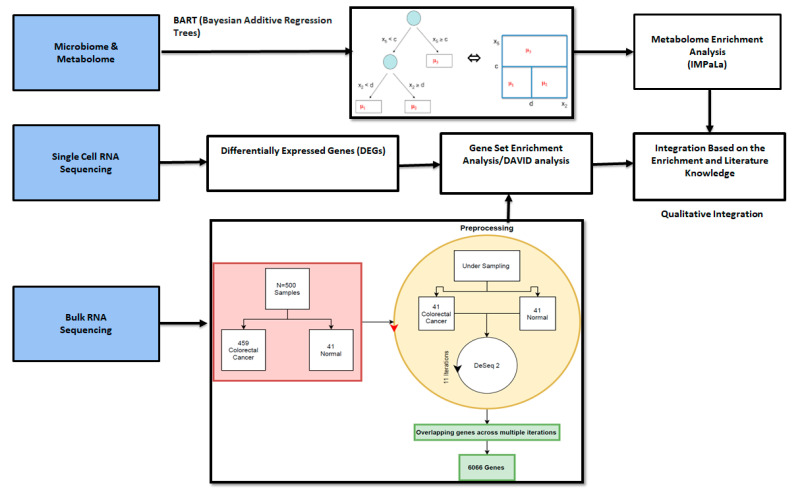
Diagrammatic representation of the qualitative integration of different methods and omics data sets to identify markers for colorectal cancer using metabolomics, transcriptomics (single-cell RNA and bulk RNA) and microbiome data sets. Gene set enrichment was used to identify associations between the genes and metabolites.

We used existing public datasets (Table 2) to identify potential mechanisms and interactions between microbiome, metabolome and genes for CRC. A workflow of all the methods and processes are described in Figure 7.

The R package “bartMachine” [86] is a Bayesian model characterised by the sum of regression trees. Consider an input *x* = (*x*_1_, ⋯, *x_p_*) with “*p*” predictors, the prediction label “*y*” is modelled as *y* = *f*(*x*) + ε, ε∼N(0, 2), where *f*(*x*) = $\overset{m}{\underset{k=1}{\sum }}T_{k}\left(x\right)$, and *T_k_*(*x*) represent regression trees.

Given an input *x*, *T_k_* assigns *x* to one of the terminal node values which is consistent with the rules used to split the predictors in *x* in the tree, and the prediction target is obtained by summing all *T_k_*(*x*), *k* = 1, ⋯, *m* from the *m* trees, plus some Gaussian noise.

Tree-based machine learning methods, such as decision tree and random forest, typically directly learn from the data the partition and the terminal node values. For the case of the BART method, these are assigned priors, and inference is drawn from the posterior distribution of the sum of trees given the data observed. One distinct feature of BART is the specification of the priors. First, as in many Bayesian machine learning methods, the prior is chosen to regularise the fit so that individual tree effect is small and no single tree dominates the prediction, a property shared by gradient boosted trees (GBM); however, unlike GBM, the number of trees m required in BART is often much smaller—studies have shown that BART performs well with no more than 200 trees across a variety of datasets [87] and in some cases the performance improvement is only marginal for using more than 50 trees [86].

Second, data-informed priors for terminal node values and observation variance are used are used to ensure that they do not deviate much from what the training data entails, a desirable property when external information is unavailable. The posterior distribution of sum-of-trees given data is simulated through the backfitting MCMC algorithm [87]. Parameter estimates and predictions can then be obtained from the posterior. Additionally, the uncertainty of the estimates can be quantified through Bayesian credible intervals. With some modification to the backfitting algorithm, the BART probit model for (binary) classification: *P*(*Y* = 1|*x*) = Φ(*f*(*x*)) can be used.

The variable importance was assessed by the fraction of times each variable is used to split the nodes in the posterior sum of trees, called “inclusion proportion”. Variables that appear frequently in the trees, hence higher inclusion proportion, are likely to be important. Significance was assessed by local thresholding [86], that is, a variable will be selected if its inclusion proportion exceeds the 0.95 quantile of its null distribution obtained by permuting the response. The interaction effect was estimated by counting the number of times a set of variables appear together in a downward path of the tree from the root node to a terminal node [86]. When the number of trees m is large, the flexibility offered by so many trees means that inclusion of irrelevant variables is unlikely to significantly degrade the fit, and so these variables could appear frequently in the trees, resulting in spurious interactions and less effective variable selection. Therefore, by limiting the number of trees to be small and forcing the variables to compete to enter into the model, BART can be an effective method for determining variable importance and interaction effect.

Metabolic enrichment analysis was conducted using IMPaLA, a publicly available web platform, that has been developed for integrated pathway analysis of metabolomics data alongside gene expression or protein abundance data [12]. It performs an over-representation or enrichment analysis with user-specified lists of metabolites and genes using over 3000 preannotated pathways from 11 databases. It provides pathway over-representation and enrichment analysis functionality with user-specified lists of genes/proteins and/or metabolites, generally termed physical entities.

We analysed the differential expression of genes involved in colon adenocarcinoma using. The TCGA-COAD (The Cancer Genome Atlas—Colon Adenocarcinoma) mRNASeq level 3 raw count data [14] generated by the UNC version 2 analysis pipeline. The inspected 20,532 gene loci out of which 29 were hypothetical loci and 500 samples (Colon adenocarcinoma: 459 and Normal: 41). We used DESeq2 to investigate the RNAseq raw count and identify differentially expressed genes [88]. DESeq2 reduces the number of genes tested by removing the genes unlikely to be significantly differentially expressed prior to testing and performs gene-level quality control. The *p*-values are attained by the Wald test and are corrected for multiple testing using the Benjamini and Hochberg method. The number of genes, in the output table of DESeq analysis with the significance cutoff set to 0.1, was the same as the original (20,532). As unbalanced class distribution of labels (Colon adenocarcinoma: 459 and Normal: 41) can affect predictive performance, specifically for minority class [89], we performed under sampling of the data [90]. We chose 41 colon adenocarcinoma samples and 41 normal samples iteratively (not repeating the samples) and analysed them using DESeq2. We then combined the results by taking the union of the list of genes in the resultant table from each iteration. We subset the results iteratively by decreasing the significance cut off from 0.05 to 0.0001 (0.05, 0.01, 0.001, 0.0001), which resulted in 12,290 genes with *p* adjusted < 0.001. We then selected the overlapping genes across all the iterations, 6066 genes.

We used Recursive Feature Elimination (RFE) to select a small subset of genes from a broad range of gene expression data [15]. RFE fits a model, like random forests (rfFuncs), and removes the weakest feature (or features) until the specified number of features is reached. We used the RFE via CARET [91]. Due to the unbalanced classes, we performed RFE iteratively on the undersampled data as we did earlier. We used random forest to select the subset of genes from each iteration. This results in a list of 345 genes collectively from all iterations. Taking the union of the list of genes from each iteration resulted in a list of 121 genes and out of 345 genes 76 appeared more than once among 11 iterations. For the 76 genes selected, we used Enrichr for the enrichment analysis [16]. Enrichr is an integrative web-based software application that includes new gene-set libraries, an alternative approach to rank enriched terms, and various interactive visualisation approaches to display enrichment results.

The FPKM values of the significant genes from the single-cell data for four different cell types were obtained from the Li et al. [13] and Zhang et al. [19] datasets. The list of genes used in this study can be found in Supplementary Table S1.

We used the DAVID (Database for Annotation, Visualization and Integrated Discovery) online tool [18] to find the significant pathways that are enriched for the different single cells’ significant genes lists.

The heatmaps were generated using gplots package heatmap2 function in R. The network analysis was done using NetworkAnalyst (v3.0) [91] where a tissue specific coexpression network was built with the degree filter of 1.0 on all the nodes. The seed genes are shown in red colour and are labelled while the proteins connected to the seed are shown in yellow. The blue colour nodes are the proteins involved in the metabolic processes.

We used enrichment analysis to associate and integrate genes and metabolites identified from transcriptomics and metabolomics datasets derived from diverse populations. Moreover, a literature-based validation was performed to gain insights to CRC associated pathways.

## 5. Conclusions

Our microbiome and metabolome analysis identified novel interactions related to 5-aminovalerate and cholesterol. Using bulk and single cell RNA sequencing, we identified 17 genes (e.g., *KIAA1199*, *CDH3*, *GUCA2B*, *LGALS4*, *CA7*, *NR3C2*, *ABCG2*, *AQP8*, etc.) including several metabolic genes (*HSD11B2*, *NR3C2*, *ABCG2*, *CA7*) associated with CRC pathophysiology. A metabolic enrichment analysis revealed a prominent of cholesterol pathways in CRC physiology. Finally, our qualitative integration approach catered the identification of the downregulation of *ABCG2* and *AQP8* genes, responsible for bile secretion, which directly increases the cholesterol synthesis facilitated by gut microbes.

## Figures and Tables

**Table 1:** List of the 17 genes and evidence of their involvement in regulation across normal and CRC patients.

Gene Symbol	Normal vs. CRC (Bulk RNA Seq)	Normal vs. CRC (Single-Cell RNA Seq) Epithelial Cells	Reference
*ADH1B*	Downregulated	Downregulated	[21]
*KIAA1199 (CEMIP)*	Upregulated	Upregulated	[22,23,24,25,26,27]
*CDH3*	Upregulated	Upregulated	[5,28,29]
*CA7*	Downregulated	Downregulated	[30,31]
*GUCA2B*	Downregulated	Downregulated	[32,33,34]
*ABCC13*	Downregulated	Downregulated	[35]
*ABCG2*	Downregulated	Downregulated	[36,37,38,39,40,41]
*CPNE7*	Upregulated	Upregulated	[42]
*HHLA2*	Downregulated	Downregulated	[43,44]
*CEACAM7*	Downregulated	Downregulated	[45,46]
*AQP8*	Downregulated	Downregulated	[47]
*GTF3A*	Upregulated	Upregulated	[48]
*MMP28*	Downregulated	Down regulated	[49,50]
*LGALS4*	Downregulated	Downregulated	[51,52,53,54,55]
*HSD11B2*	Downregulated	Downregulated	[56,57,58]
*CHP2*	Downregulated	Downregulated	[59]
*NR3C2*	Downregulated	Downregulated	[32,34,60,61,62]

**Table 2:** Description of the microbiome, metabolome, bulk RNA sequencing and single cell sequencing datasets.

Data Set	Features	Sample	Reference
Kim et al., 2020	16S rRNA and metabolomics	Normal (*N* = 102) vs. Colorectal cancer (*N* = 36)	[8]
Clos-Garcia et al., 2020	16S rRNA and metabolomics	Normal (*N* = 77) vs. Colorectal cancer (*N* = 99)	[11]
The Cancer Genome Atlas (TCGA) ColonAdenocarcinoma	RNA sequence	Normal (*N* = 41) vs. Colon Adenocarcinoma (*N* = 459)	[85]
Li et al., 2017	Single-cell transcriptomes	Normal (*N* = 1591 cells) vs. Colorectal cancer (*N* = 1591 cells)	[17]
Zhang et al., 2019	Single-cell transcriptomes	Normal (*N* = 160 cells) vs. Colorectal cancer (*N* = 272 cells)	[19]

## Data Availability

All the datasets are freely available. TCGA data was downloaded from https://portal.gdc.cancer.gov/projects/TCGA-COAD (accessed on 30 November 2020).

## Supplementary Materials

The following are available online at https://www.mdpi.com/article/10.3390/ijms22115763/s1.
