# Supplementary material for: Integration of the Microbiome, Metabolome and Transcriptomics Data Identified Novel Metabolic Pathway Regulation in Colorectal Cancer
Source: Int J Mol Sci. 2021 May 28;22(11):5763. doi: 10.3390/ijms22115763 (PMC8198673; doi:10.3390/ijms22115763)
Supplement: Supplementary file 1 [file ijms-22-05763-s001.zip › Supplementary Figure S1.pdf]

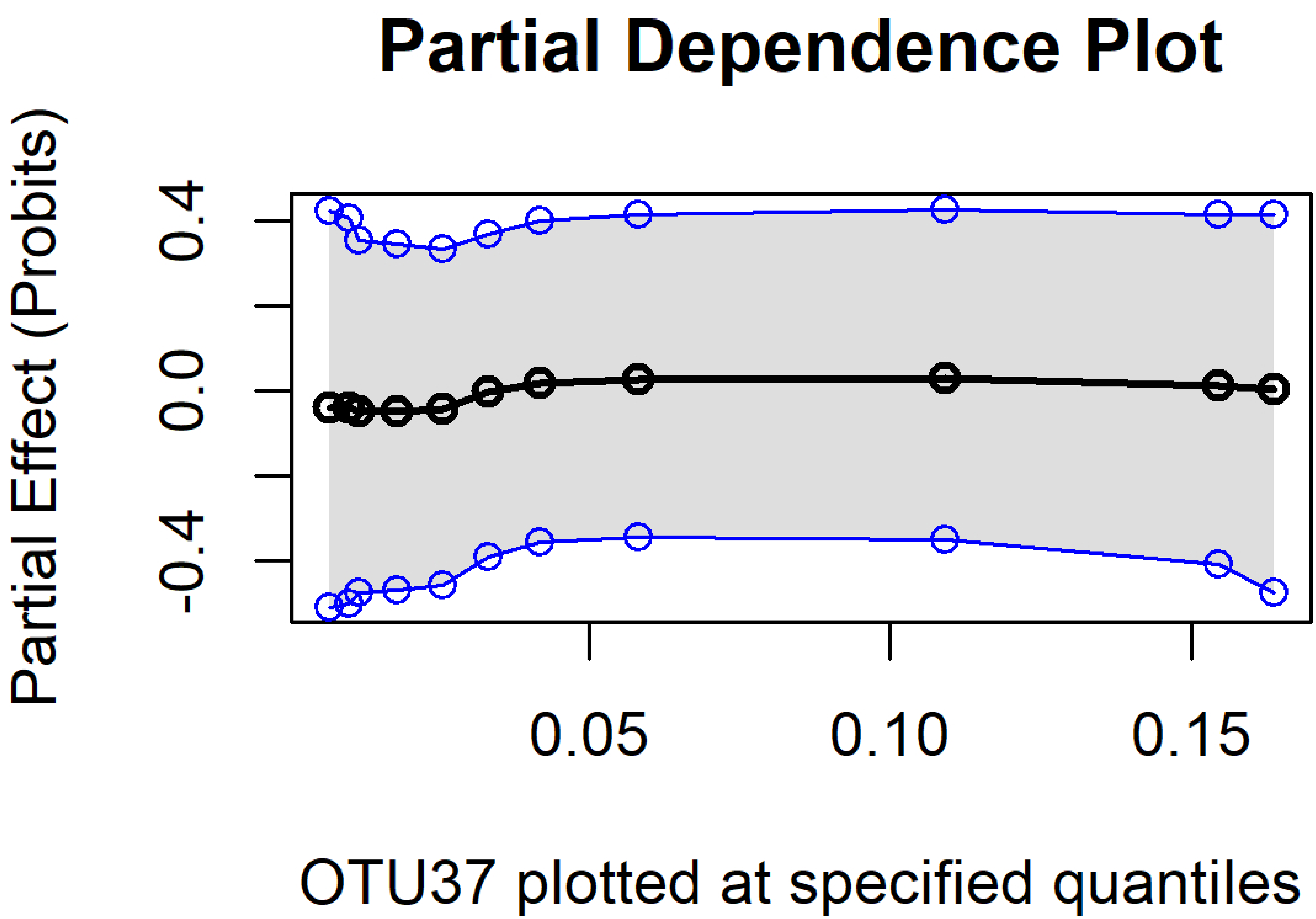
Partial Dependence Plot
Partial Effect (Probits)
0.4
0.0
-0.4
0.05
0.10
0.15
OTU37 plotted at specified quantiles

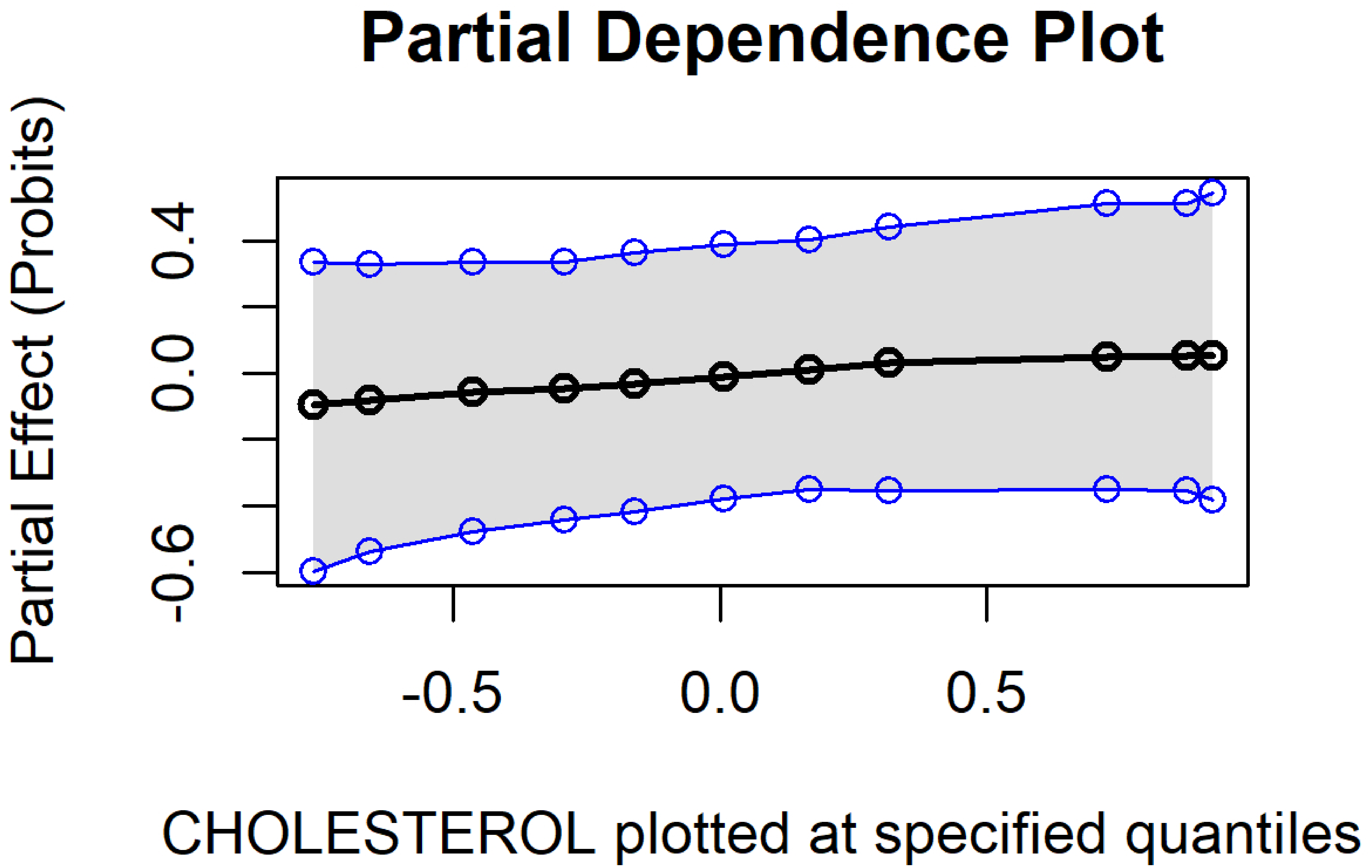
Partial Dependence Plot
Partial Effect (Probits)
0.4
0.0
-0.6
-0.5
0.0
0.5
CHOLESTEROL plotted at specified quantiles

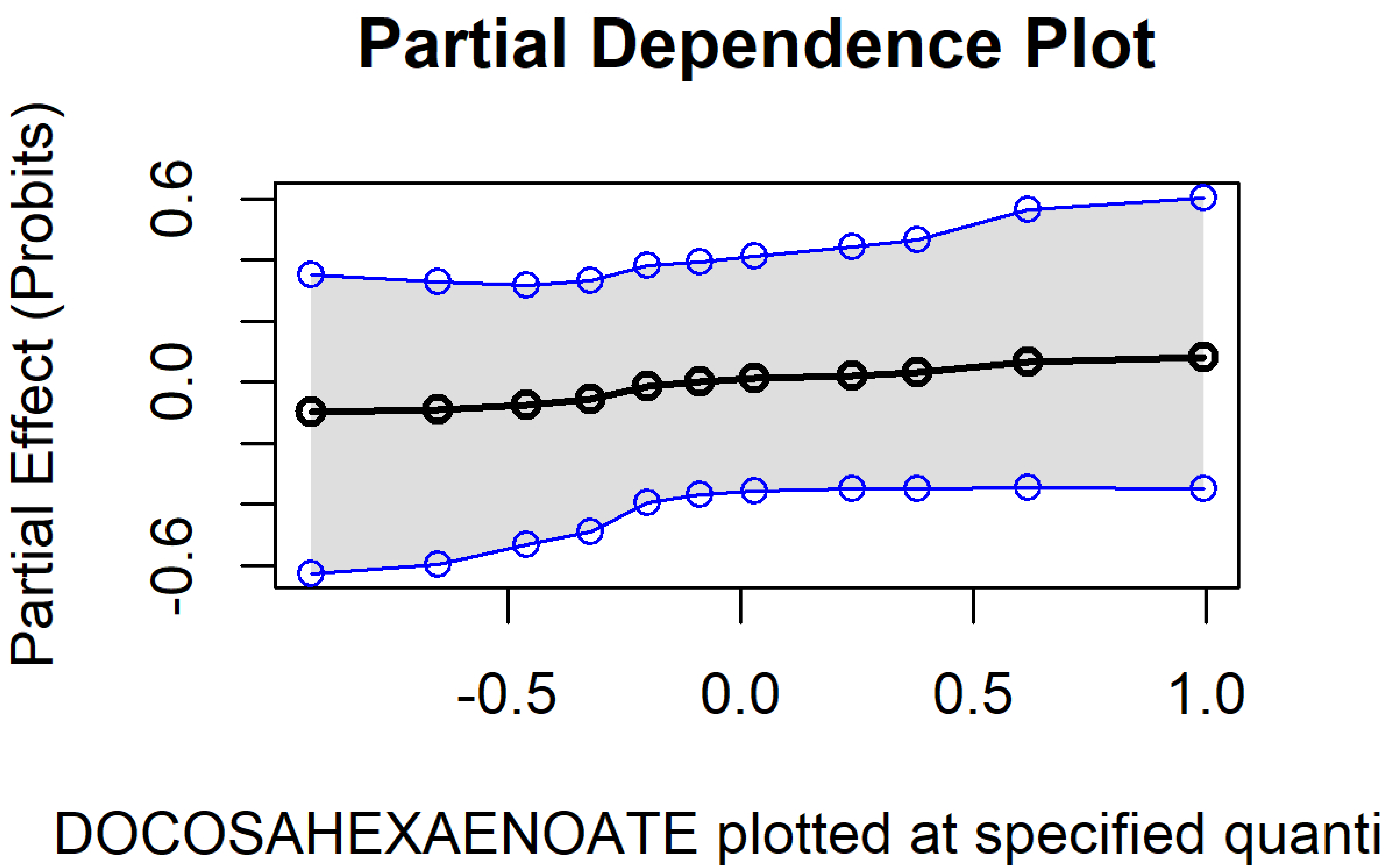
Partial Dependence Plot
Partial Effect (Probits)
0.6
0.0
-0.6
-0.5
0.0
0.5
1.0
DOCOSAHEXAENOATE plotted at specified quanti

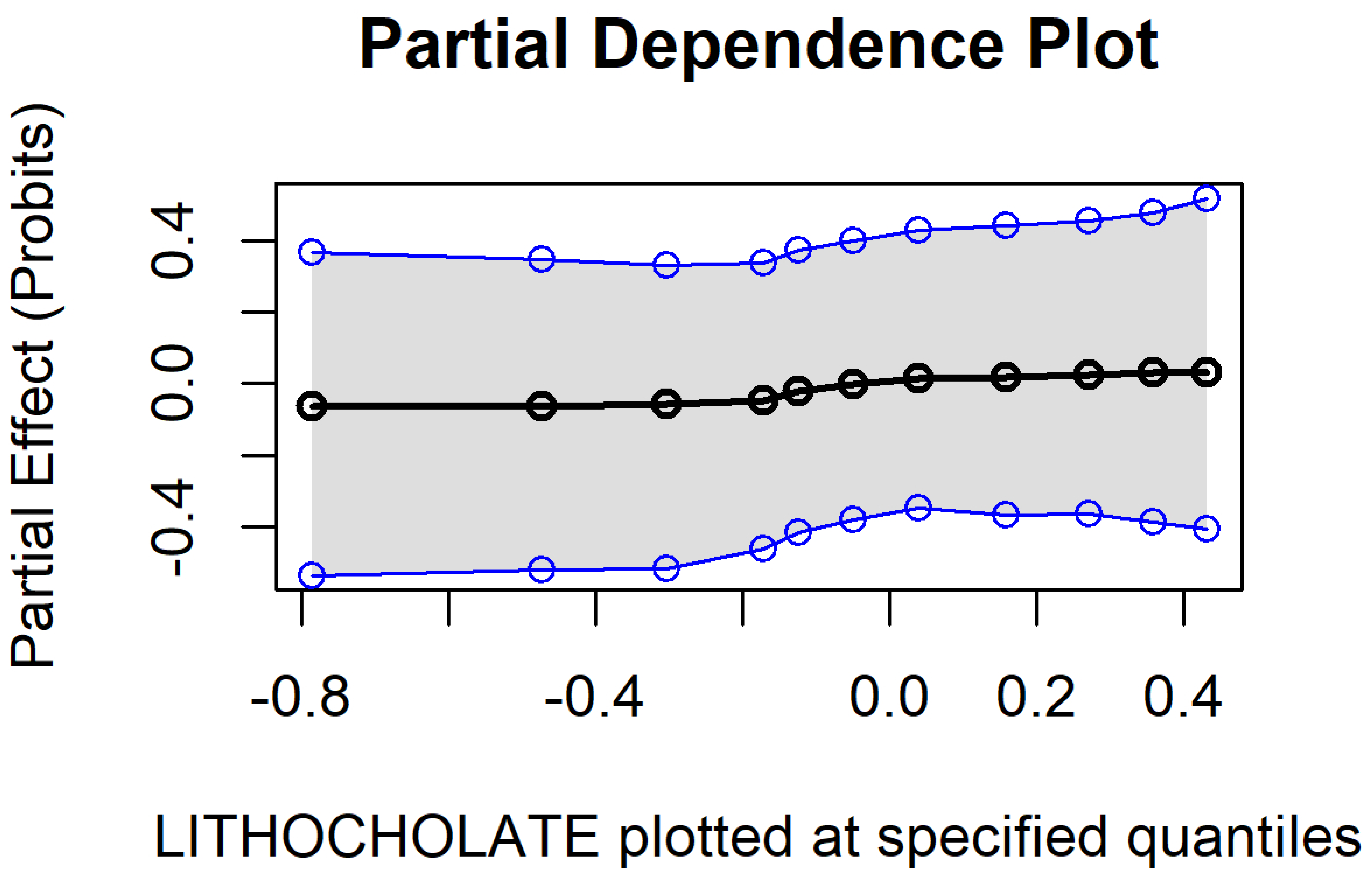
Partial Dependence Plot
Partial Effect (Probits)
0.4
0.0
-0.4
-0.8
-0.4
0.0
0.2
0.4
LITHOCHOLATE plotted at specified quantiles

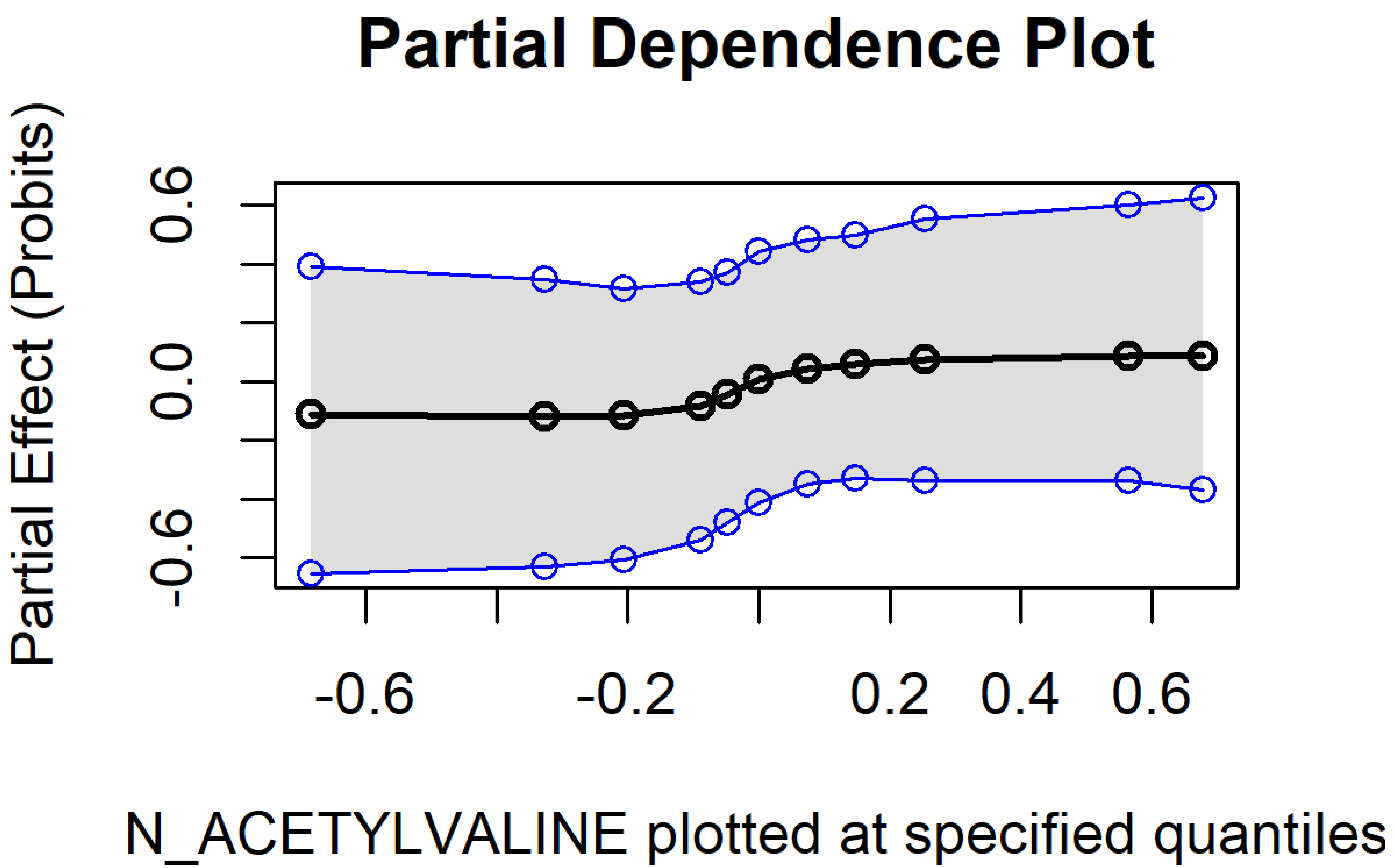

Partial Dependence Plot
Partial Effect (Probits)
0.6
0.0
-0.6
-0.6
-0.2
0.2
0.4
0.6
N_ACETYLVALINE plotted at specified quantiles

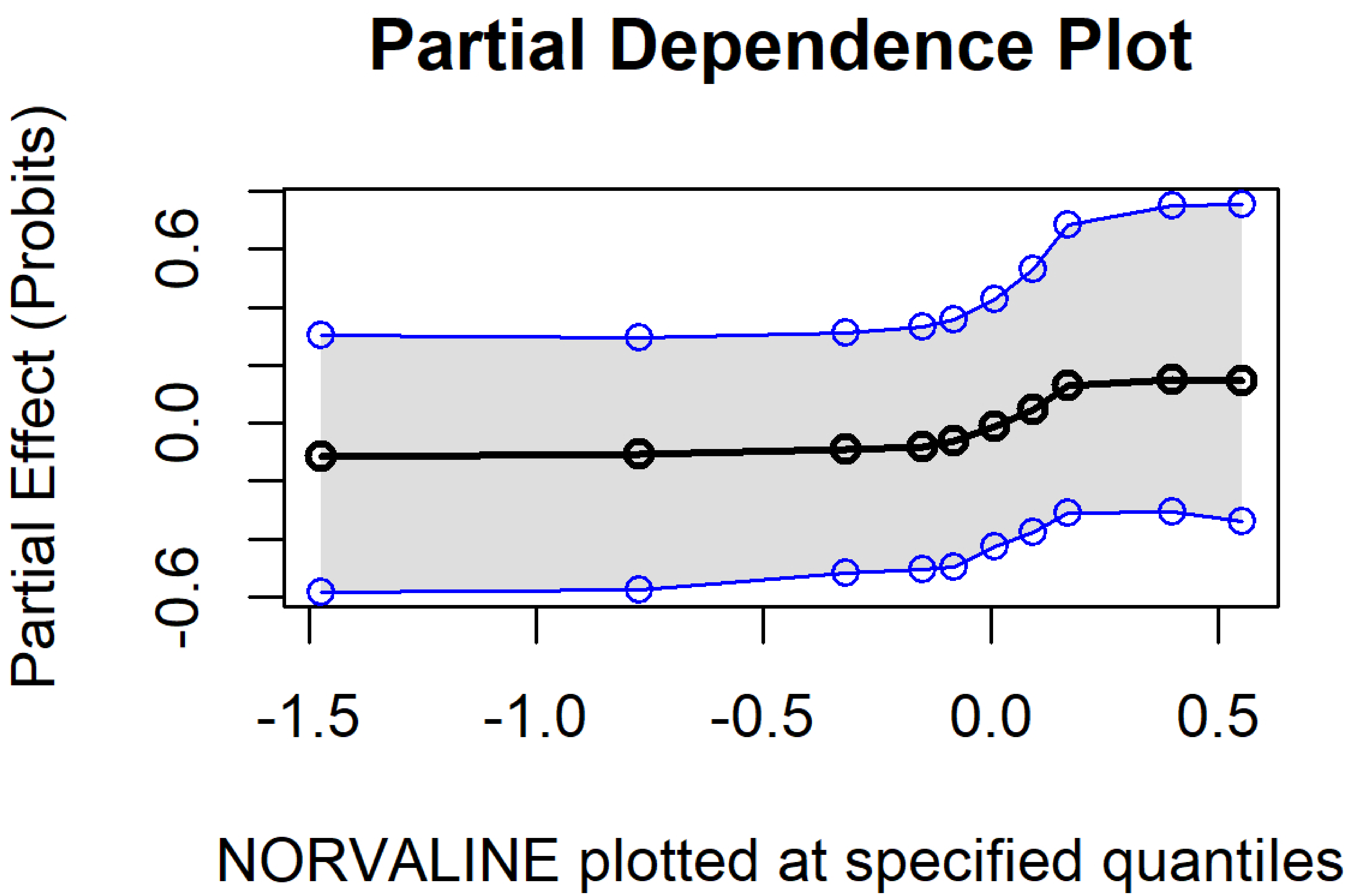
Partial Dependence Plot
Partial Effect (Probits)
0.6
0.0
-0.6
-1.5
-1.0
-0.5
0.0
0.5
NORVALINE plotted at specified quantiles

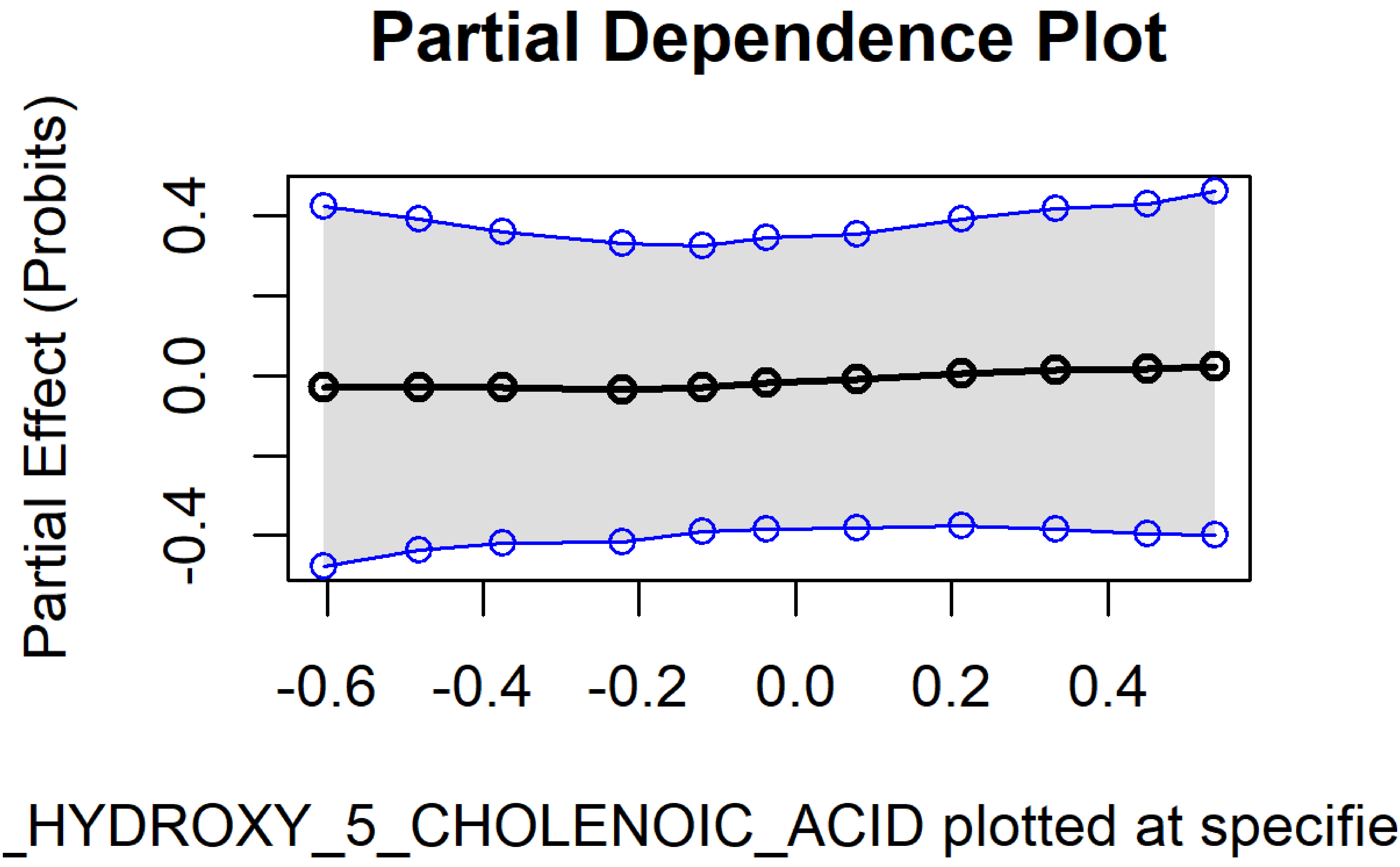

Partial Dependence Plot
Partial Effect (Probits)
0.4
0.0
-0.4
-0.6
-0.4
-0.2
0.0
0.2
0.4
_HYDROXY_5_CHOLENOIC_ACID plotted at specifie

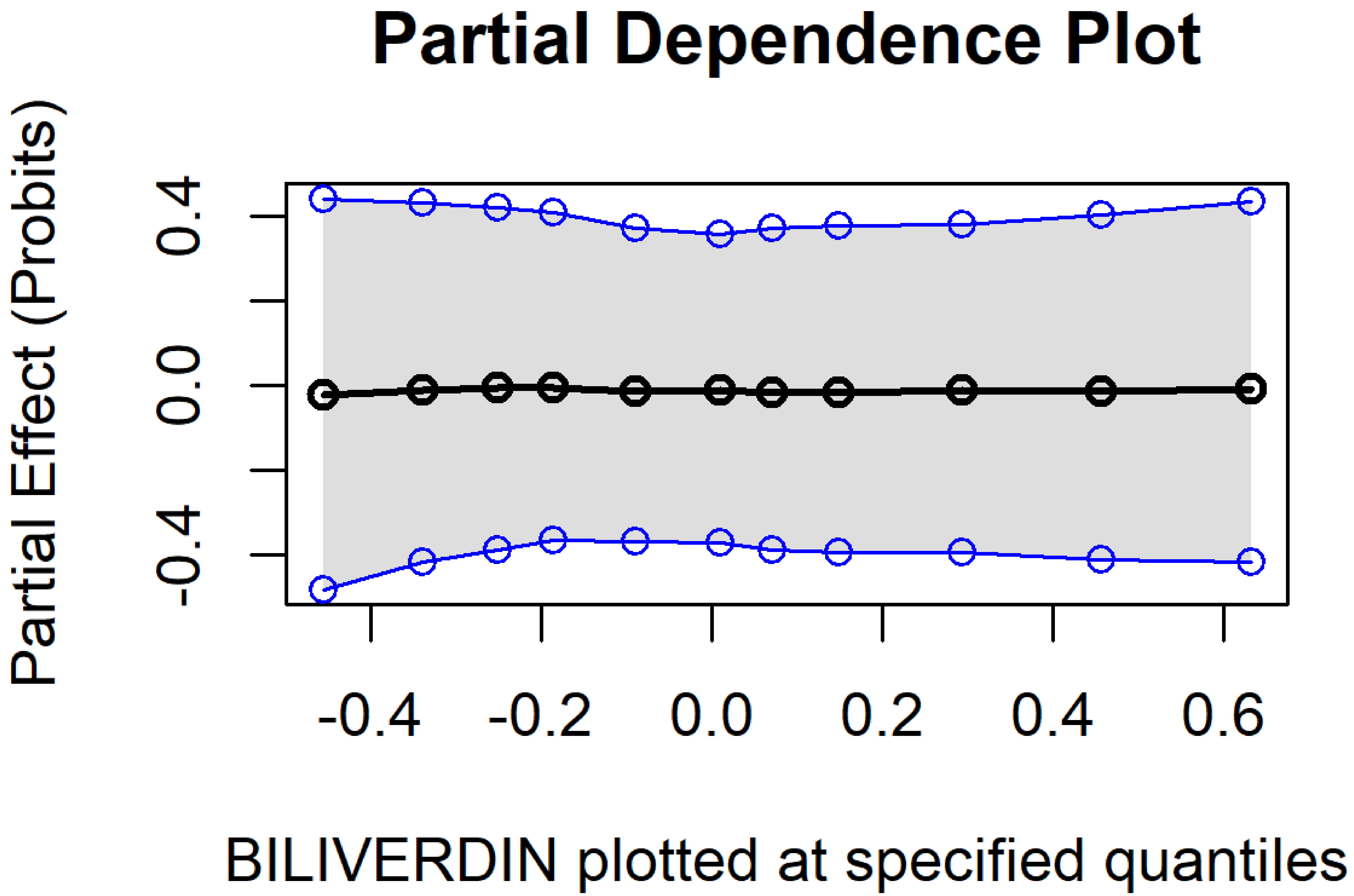

Partial Dependence Plot
Partial Effect (Probits)
0.4
0.0
-0.4
-0.4
-0.2
0.0
0.2
0.4
0.6
BILIVERDIN plotted at specified quantiles

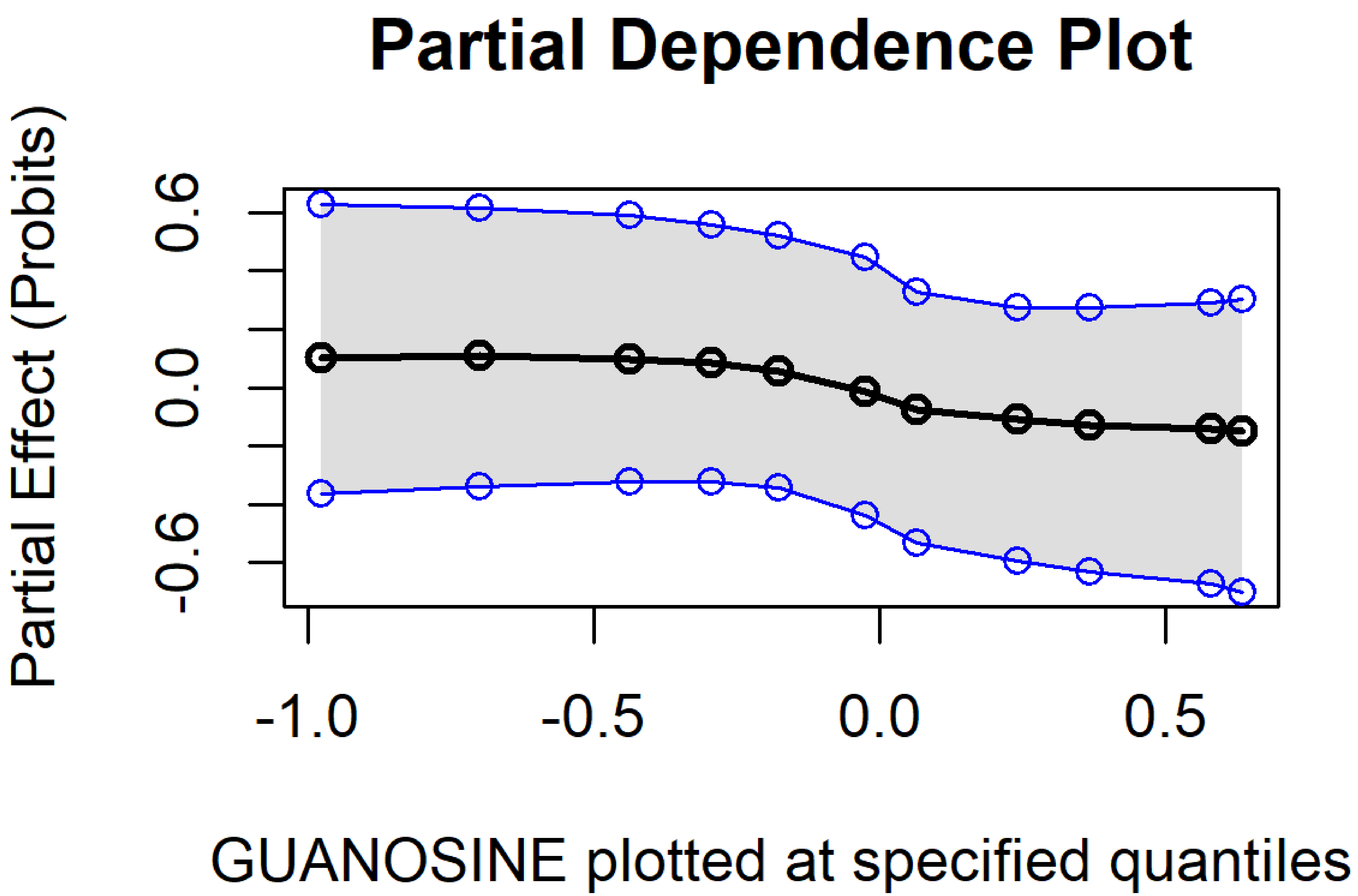
Partial Dependence Plot
Partial Effect (Probits)
0.6
0.0
-0.6
-1.0
-0.5
0.0
0.5
GUANOSINE plotted at specified quantiles

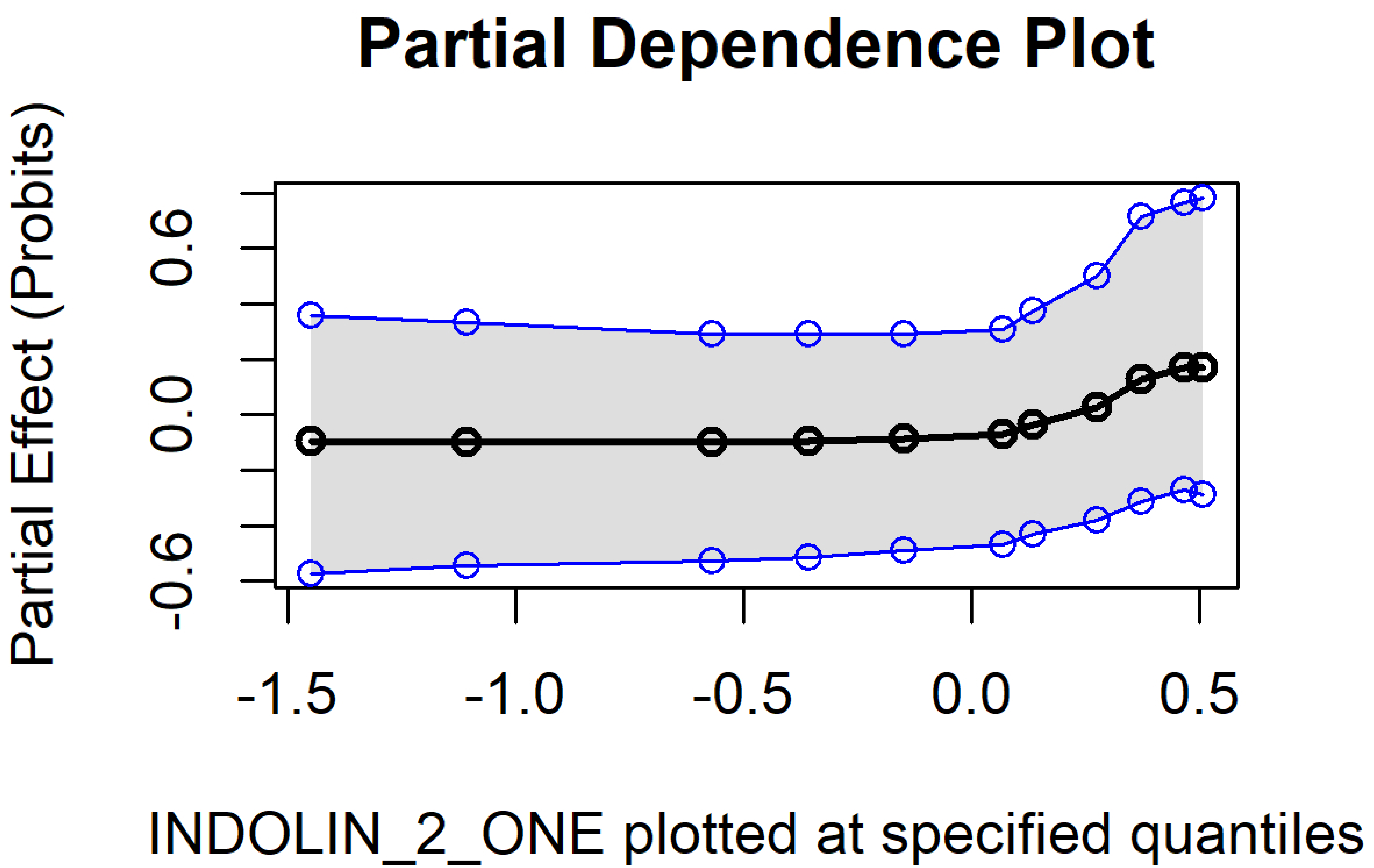
Partial Dependence Plot
Partial Effect (Probits)
0.6
0.0
-0.6
-1.5
-1.0
-0.5
0.0
0.5
INDOLIN_2_ONE plotted at specified quantiles

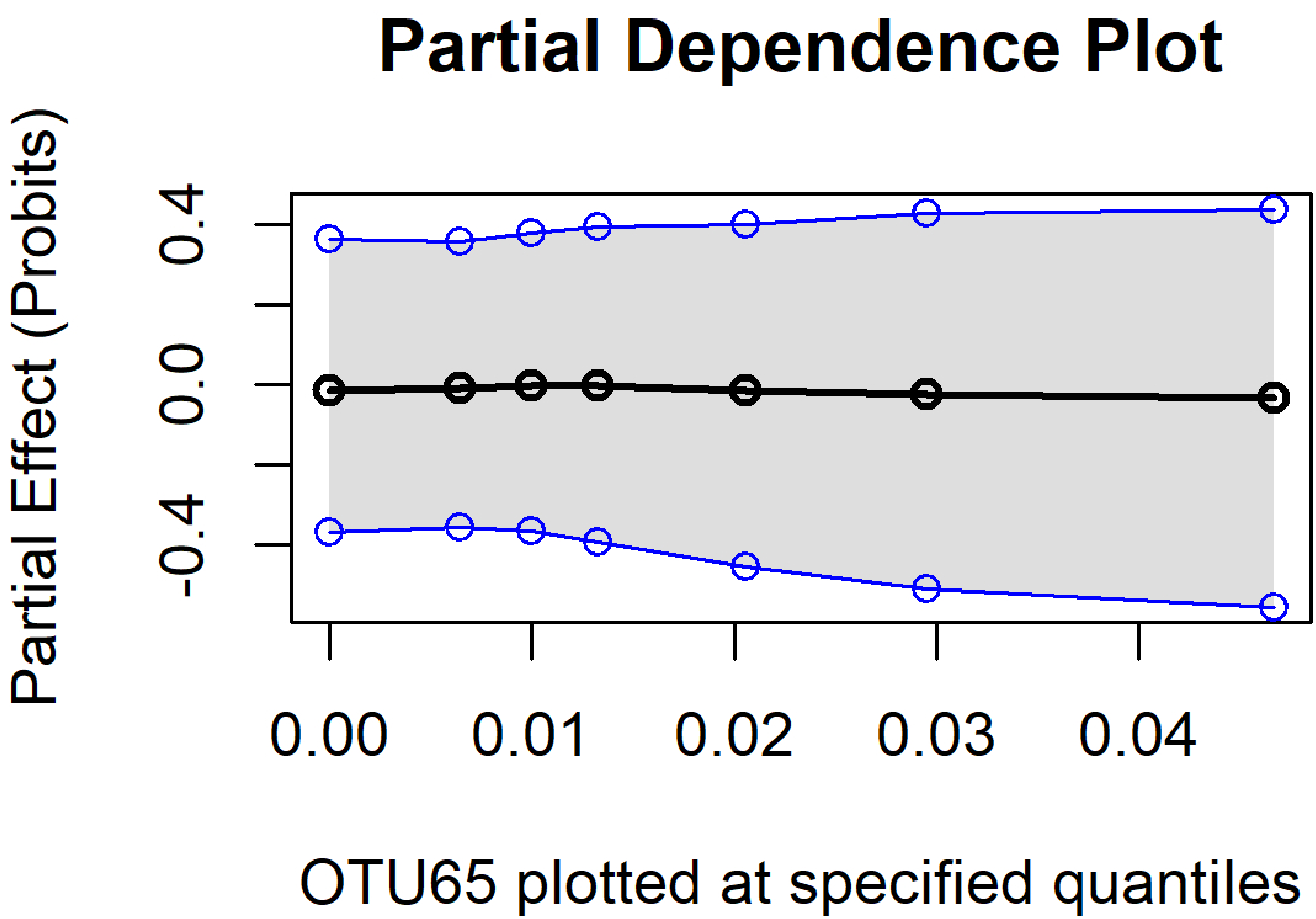

Partial Dependence Plot
Partial Effect (Probits)
0.4
0.0
-0.4
0.00
0.01
0.02
0.03
0.04
OTU65 plotted at specified quantiles
