# Supplementary material for: Integration of the Microbiome, Metabolome and Transcriptomics Data Identified Novel Metabolic Pathway Regulation in Colorectal Cancer
Source: Int J Mol Sci. 2021 May 28;22(11):5763. doi: 10.3390/ijms22115763 (PMC8198673; doi:10.3390/ijms22115763)
Supplement: Supplementary file 1 [file ijms-22-05763-s001.zip › Supplementary Table S3.pdf]

Table S3: Pairwise interactions between microbial/metabolomic features in Figure 2 and Feature 1 and corresponding feature in Feature 2 is likely associated with a change in CRC risk. Notation used: increased (+), decreased (-).

Feature 1	Feature 2	CRC Risk
5-aminovalerate (+)	Harmane (-)	+
5-aminovalerate (-)	Harmane (+)	-
5-aminovalerate (+)	1,2-Dilinoleoyl-GPC (18:2/18:2) (+)	+
5-aminovalerate (-)	1,2-Dilinoleoyl-GPC (18:2/18:2) (-)	-
5-aminovalerate (+)	1-oleoyl-2-linoleoyl-GPC (18:1/18:2) (+)	+
5-aminovalerate (-)	1-oleoyl-2-linoleoyl-GPC (18:1/18:2) (-)	-
5-aminovalerate (+)	Guanosine (-)	+
5-aminovalerate (-)	Guanosine (+)	-
5-aminovalerate (+)	N-acetylvaline (+)	+
5-aminovalerate (-)	N-acetylvaline (-)	-
5-aminovalerate (+)	Indolin-2-one (+)	+
5-aminovalerate (+)	arabonate/xylonate (-)	+
5-aminovalerate (+)	Glycerate (-)	+
5-aminovalerate (-)	Glycerate (+)	-
5-aminovalerate (+)	N-formylmethionine (-)	+
5-aminovalerate (-)	N-formylmethionine (+)	-
5-aminovalerate (+)	Adlercreutzia (OTU2) (+)	+
5-aminovalerate (-)	Adlercreutzia (OTU2) (-)	-
5-aminovalerate (+)	Pyridoxamine (-)	+
5-aminovalerate (-)	Pyridoxamine (+)	-
Indolin-2-one (+)	1,2-Dilinoleoyl-GPC (18:2/18:2) (+)	+
Indolin-2-one (-)	1,2-Dilinoleoyl-GPC (18:2/18:2) (-)	-
Indolin-2-one (+)	docosahexaenoate (DHA; 22:6n3) (+)	+
Indolin-2-one (-)	docosahexaenoate (DHA; 22:6n3) (-)	-
arabonate/xylonate (+)	1,2-Dilinoleoyl-GPC (18:2/18:2) (-)	-
arabonate/xylonate (-)	1,2-Dilinoleoyl-GPC (18:2/18:2) (+)	+
