# Supplementary material for: Integration of the Microbiome, Metabolome and Transcriptomics Data Identified Novel Metabolic Pathway Regulation in Colorectal Cancer
Source: Int J Mol Sci. 2021 May 28;22(11):5763. doi: 10.3390/ijms22115763 (PMC8198673; doi:10.3390/ijms22115763)
Supplement: Supplementary file 1 [file ijms-22-05763-s001.zip › Supplementary Table S4.pdf]

Table S4: Pairwise interactions between microbial/metabolomic features in Figure 3(b). An AND relation between a feature in Feature 1 and corresponding feature in Feature 2 is likely associated with a change in CRC risk. Notation used: increased (+), decreased (-).

Feature 1	Feature 2	CRC Risk
ChoE(20:4) (+)	Staphylococcus (OTU7517) (+)	+
ChoE(20:4) (-)	Staphylococcus (OTU7517) (-)	-
ChoE(20:4) (+)	Blautia (OTU14967) (-)	+
ChoE(20:4) (-)	Blautia (OTU14967) (+)	-
ChoE(20:4) (+)	Roseburia (OTU9785) (+)	+
ChoE(20:4) (-)	Roseburia (OTU9785) (-)	-
Staphylococcus (OTU7517) (+)	Roseburia (OTU9785) (+)	+
Staphylococcus (OTU7517) (-)	Roseburia (OTU9785) (-)	-
Blautia (OTU14967) (+)	Unknown genus of Lachnospiraceae (OTU17213) (+)	-
Blautia (OTU14967) (-)	Unknown genus of Lachnospiraceae (OTU17213) (-)	+
