# Supplementary figures and images for: Integration of the Microbiome, Metabolome and Transcriptomics Data Identified Novel Metabolic Pathway Regulation in Colorectal Cancer
Source: Int J Mol Sci. 2021 May 28;22(11):5763. doi: 10.3390/ijms22115763 (PMC8198673; doi:10.3390/ijms22115763)

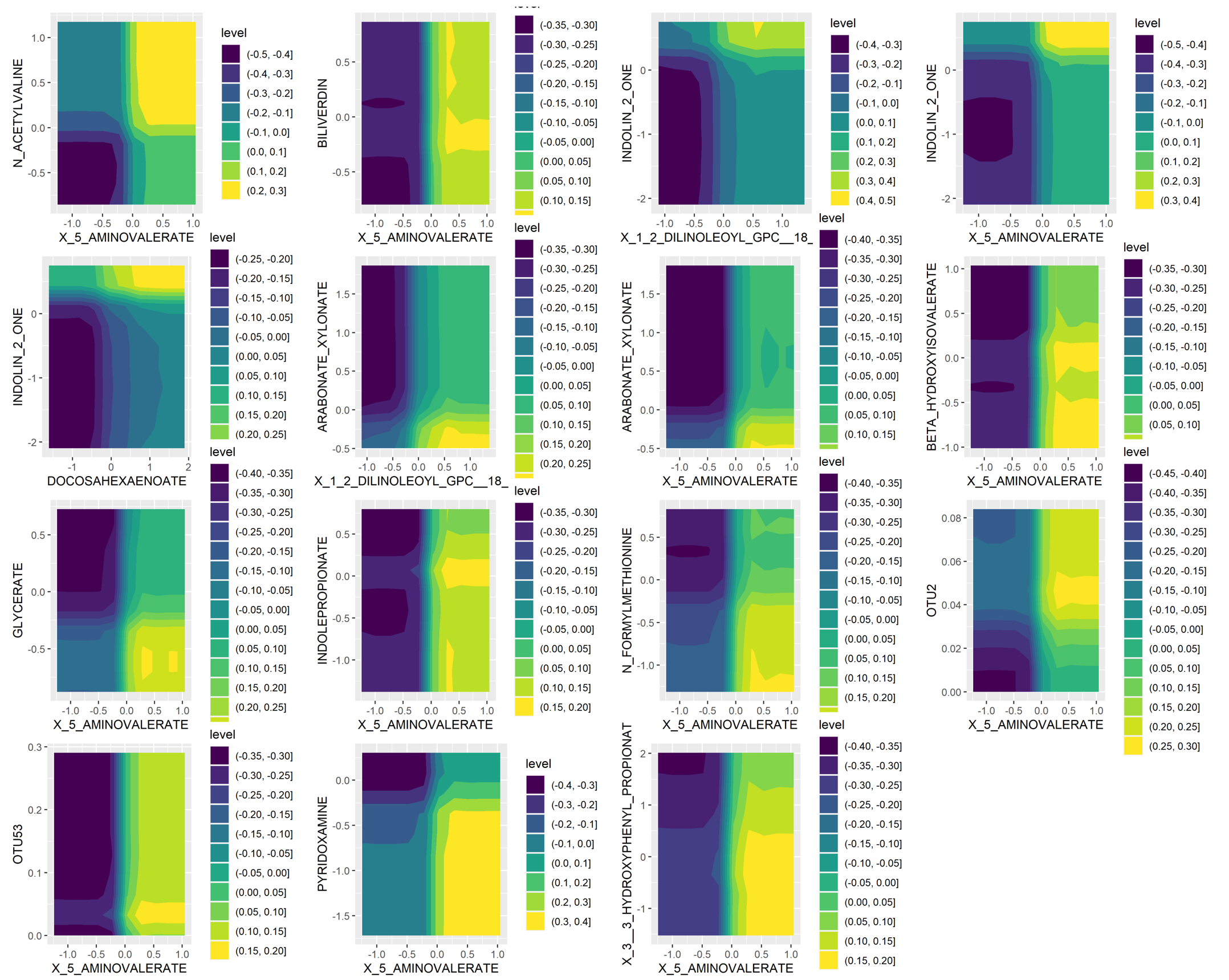

Supplement: Supplementary file 1 [file ijms-22-05763-s001.zip › Supplementary Figure S2.pdf]
